# Rare Case of Spina Bifida

**Published:** 1861-05

**Authors:** Chas. M. Anderson

**Affiliations:** Minneapolis, Minn.


CHICAGO

MEDICAL JOURNAL.

VOL. IV.] MAY, 1861. [NO. 5.

### ORIGINAL COMMUNICATONS.

### RARE CASE OF SPINA BIFIDA.

BY CHAS. M. ANDERSON, M.D., Minneapolis, Minn.

On the 16th of Nov. 1858, Mrs. N. was delivered of her second child. Nothing unusual was experienced during pregnancy, except a slight dyspepsia about the fourth and fifth months. The labor and presentation were natural. On examination of the child (a female), I found a Spina Bifida. The tumor was about the size of a hen's egg—somewhat pear-shaped, with the smaller end attached by a good sized peduncle. It did not differ from ordinary spinal tumors except as to locality. It was situated *between the occipital protuberance and the foramen magnum*—apparently emerging from a fissure in the posterior margin of the latter.

The child lived five weeks. It gradually became emaciated and died of vital exhaustion. The tumor remained nearly the same in size and appearance until death. The parents were adverse to any treatment which might look like "experiment."

Consequently little or nothing was done. No *post mortem* was allowed.

In other respects the child was well formed. The head and spine appeared natural, and for several weeks it took its food with a good appetite. I have examined the record of cases in the different books and periodicals at my command, and find this to be a very unusual case. Hence I have deemed it desirable to place it on record.

Prof. Brainard, in an excellent article on Spina Bifida in this *Journal* for Sept. 1859, speaks of a little girl, aged about six years, with Spina Bifida situated at "the occipital protuberance," "of the size and shape of a pear." This is about the only similar case that I find any notice of.

Rokitansky, however, has this remark: "The most common situation of Spina Bifida is the lower dorsal and lumbar region. Fissure of the sacral vertebræ is more rare: sometimes it occurs in two places together, and then usually one fissure is in the *neck*, and combined with hemicephalus, while there is another in the lumbar region."—*Vol.* 3, *p.* 177, *of Path. Anat.*

---

The above is certainly a rare case, but it is not alone in the science, several others being found scattered through different books, journals and transactions of societies. It does not appear that the death resulted from the malformation, although a deficiency of parts of the nervous centres sometimes coexists, with *spina bifida* incompatible with prolonged life. In regard to treatment it is doubtful if a case can be found on record of such a tumor in that situation treated in any way. If the child had survived I should have had no hesitation in advising the injection of iodine according to the rules laid down in the publication referred to by Dr. Anderson.

As this method is still regarded by many as experimental, I take the liberty of publishing a private letter from Dr. Ellinwood, of this city, a young gentleman now completing his medical studies in Paris, which indicates the estimation in which it is held in France. Some of the cases treated in this country have been either unsuitable for any operation, or the operations have been repeated unnecessarily and performed imperfectly. There is indeed no class of cases requiring more careful discrimination and no operation demanding more careful attention to details and perfect adaptation of instruments. In the hands of M. Nelaton there is little danger of its losing credit. [B.]

PARIS, March 26th, 1861.

PROF. BRAINARD—*Dear Sir* :—It was with much pleasure I witnessed yesterday your operation for Spina Bifida performed by M. Nelaton, as "Brainard's Method," in his crowded amphitheatre. The distinguished Professor made some very interesting remarks on the disease; adopts your excellent memoir on the subject, and concludes that your operation—Iodine Injections—offers the greatest probability of success.

M. Viard is the author of an elaborate paper on Spina Bifida which was read before the "Société de Chirurgie" in November last, and published in the *Gazette des Hopitaux*. He reports your cases and many others treated by your method, showing very satisfactory results.

Very respectfully, C. N. ELLINWOOD.

